# Acute lower limb compartment syndrome after Cesarean section: a case report

**DOI:** 10.1186/1752-1947-5-161

**Published:** 2011-04-22

**Authors:** Julia C Radosa, Marc P Radosa, Marc Sütterlin

**Affiliations:** 1 Department of Gynecology & Obstetrics, University Medical Center Mannheim, University of Heidelberg, Theodor-Kutzer Ufer 1-3, D-68167 Mannheim, Germany; 2 Department of Gynecology & Obstetrics, Jena University Hospital, Jena, Germany

## Abstract

**Introduction:**

Acute compartment syndrome of the lower limb is a rare but severe intra- and post-partum complication. Prompt diagnosis is essential to avoid permanent functional restriction or even the loss of the affected limb. Clinical signs and symptoms might be nonspecific, especially in the early stages; therefore, knowledge of predisposing risk factors can be helpful.

**Case presentation:**

We present the case of a 32-year-old Caucasian woman with acute post-partum compartment syndrome.

**Conclusion:**

Acute compartment syndrome is an important differential diagnosis for the sudden onset of intra- or post-partum lower-limb pain. Predisposing factors for the manifestation of acute compartment syndrome in an obstetric environment are augmented intra-partum blood loss, prolonged hypotensive episodes and the use of oxytocin to support or induce labor because of its vasoconstrictive properties. Treatment is prompt surgical decompression by performing fasciotomy in any affected muscular compartments.

## Introduction

Acute limb compartment syndrome (ACS) is a condition in which increased pressure within a closed musculofascial compartment compromises blood circulation and biomechanical function. There are several etiologies of ACS. ACS may occur after significant trauma, for example, long-bone fractures. Other forms of injury which cause soft tissue damage, such as crush injuries, severe thermal burns and bleeding diathesis are known causes as well. Less frequently ACS may occur in a non-traumatic setting, such as in post-ischemic reperfusion, in revascularization procedures, after the application of vasoconstrictive therapeutic agents or in anesthesia-induced hypotension [1]. An iatrogenic cause, prolonged limb compression occurring in surgical procedures carried out with the patient in the lithotomy position (the Lloyd-Davies position), has been described in the literature [2].

Pathophysiologically, the expansion of tissue in a closed muscle compartment in ACS leads to an increase in pressure, which subsequently causes compression of thin-walled veins within that compartment [3]. As a result, venous outflow decreases and venous and arterial intra-vasal pressure increase, which causes diminished perfusion of the affected compartment [4]. The consequences of this insufficient perfusion are nerve and muscle ischemia. Muscle infarction and lasting nerve damage will occur if prompt surgical decompression is delayed.

ACS is diagnosed on the basis of clinical evaluation. In cases with an atypical or unclear clinical presentation, the invasive measurement of compartment pressure might be helpful [5]. Continuous monitoring of tissue oxygen saturation using near infrared spectroscopy has been described as particularly helpful in the diagnosis of ACS, because a sudden decrease in tissue oxygen saturation might be a first warning sign [6].

Severe pain, which appears to be out of proportion in relation to the apparent injury, is often the major clinical sign of ACS. Pain on passive stretch of the muscles and tenseness are further clinical signs frequently encountered in ACS. In the late stage of ACS, sensory deficits, paresthesias, muscle weakness, paralysis, pallor and pulselessness are typical features [7]. Definitive treatment for patients with ACS consists of decompression of the affected compartment by performing surgical fasciotomy.

## Case presentation

A 32-year-old primigravida Caucasian woman came to our department at 38 weeks and four days of gestation with spontaneous onset of labor and rupture of membranes after an uncomplicated pregnancy. The patient received an oxytocin infusion (Oxytocin 10 I.E., Oxytocin Hexal, Hexal AG, 83607 Holzkirchen, Germany) in 250 ml of 0.9% NaCl for labor stimulation, and an epidural catheter for anesthesia was applied. Seven hours after the patient was admitted to the hospital, we opted to perform a Cesarean section because of failure to progress in the first stage of labor and a non-reassuring fetal heart rate during continuous cardiotocography monitoring. A Cesarean section was performed without intra-operative complications, and a healthy male infant was delivered.

Five hours after the intervention and the patient's readmission to the hospital ward, the patient complained of a spasm-like pain in her right lower leg. An examination revealed mild tenseness and swelling of the right pretibial region. A Doppler ultrasound examination performed to exclude deep venous thrombosis showed no remarkable findings. Hence analgesic treatment with paracetamol (1000 mg oral) and piritramide (15 mg in 250 ml of 0.9% NaCl intra-venous) was started. However, the patient's symptoms did not improve, and she was re-examined one hour after the onset of her initial symptoms. The tenseness and swelling had now progressed, and measurement of her calf diameters showed a difference of 1 cm between the right and left calves. No sensory deficit was noted, her pedal pulses were palpable on both sides and her tendon reflexes were symmetrical. However, a discrete weakness of flexion of the right foot was observed, which led to the clinical suspicion of ACS. The patient was taken to the surgical theater, and ACS of the anterior tibial compartment was found during surgical exploration. A fasciotomy without resection of muscular tissue was subsequently carried out. After the surgical intervention, the patient reported immediate relief of the initial symptoms. Secondary wound closure of the open fasciotomy was performed within the following 10 post-operative days using a shoelace technique, and after 11 days the patient could be released to out-patient care. Moderate weakness of great toe extension and flexion in the right ankle joint, still present at the time of discharge, continued to be treated with physical therapy in our out-patient department. A full functional recovery of the limb was achieved within 15 days of discharge.

## Discussion

ACS is a complication which usually occurs in the setting of a traumatic injury or as a post-operative complication after prolonged surgical procedures. Several risk factors for the manifestation of ACS have been described, including prolonged hypotensive episodes, fluid deficit, treatment with vasoconstrictive agents, vascular occlusion, lying in the lithotomy position, prolonged surgery time, the use of compressive bandages and obesity [8]. In obstetrics, ACS is a relatively rare complication: Its prevalence has been estimated to be within two per 10,000 births [9].

Most ACS in obstetric patients described in the literature occurred in the setting of Cesarean delivery [8,9]. Interestingly, in all of these cases, the Cesarean section was initially complicated by a massive blood loss because of disseminated intra-vascular coagulopathy. ACS has also been reported following vaginal delivery [10]. In these cases, ACS occurred in the setting of a retained placenta leading to hypovolemic shock due to extensive blood loss.

Most authors consider a combination of factors to be causes of post-partum ACS, such as augmented intra-partum blood loss, prolonged hypotensive episodes and the use of oxytocin to support or induce labor, owing to its vasoconstrictive properties [11]. Several of these described risk factors were present in our patient. We used oxytocin to support labor, and the patient underwent epidural anesthesia with the possibility of an unnoticed hypotensive episode, since we did not monitor the patient's blood pressure continuously and the delivery was performed by Cesarean section. It is difficult to further clarify the role of these factors and their contribution to the development of ACS in our patient *ex post facto*. However, the knowledge of these predisposing factors for post-partum ACS can be a valuable help in correctly interpreting the often unspecific early clinical symptoms of this entity, since diagnostic delay might jeopardize the therapeutic outcome.

## Conclusion

ACS is a rare but severe complication which can occur during and after labor. Because the functional outcome after ACS is directly related to undelayed surgical intervention, it is essential to be aware of ACS in the differential diagnosis in patients with severe intra- and post-partum lower-limb pain.

## Competing interests

The authors declare that they have no competing interests.

## Consent

Written informed consent was obtained from the patient for publication of this case report and any accompanying images. A copy of the written consent is available for review by the Editor-in-Chief of this journal.

## Authors' contributions

JCR and MPR contributed equally to the preparation of this manuscript. MS supervised the clinical care of the patient and the preparation of this manuscript as the medical head of our department. All authors read and approved the final manuscript.
